# Thermal and Mechanical Performance of Steel Slag and Graphite Modified Concrete with a Comparative Engineering Cost Assessment

**DOI:** 10.3390/ma19020306

**Published:** 2026-01-12

**Authors:** Huidong Shan, Yun Cong, Yan Zhang, Zhuowen Yang, Hongbo Tan

**Affiliations:** 1 State Key Laboratory of Silicate Materials for Architectures, Wuhan University of Technology, Wuhan 430070, China; shanhuidong1234@163.com (H.S.); zhuoweny@whut.edu.cn (Z.Y.); 2 Zhoushan Power Supply Company, State Grid Zhejiang Electric Power Co., Ltd., Zhoushan 316000, China; congyun@zs.zj.sgcc.com.cn; 3 State Grid Electric Power Research Institute Co., Ltd., Beijing 100069, China; 19157424064@163.com

**Keywords:** thermally conductive concrete, thermal conductivity, mechanical properties, value engineering

## Abstract

**Highlights:**

**What are the main findings?**

- High thermal conductivity concrete was developed using steel slag, graphite powder and granules, and quartz sand.
- A 10% graphite particle replacement increased 28-day thermal conductivity by 106.16%.
- Compressive strength was maintained at 25.1 MPa with good mechanical performance.
- Value coefficient improved by 18.31% compared to conventional C25 concrete.

**What are the implications of the main findings?**

- Enhanced heat dissipation efficiency for cable duct encasement systems.
- Improved thermal stability and operational safety of underground power cables.
- Provided material design basis for power engineering and energy-saving construction.

**Abstract:**

The heat generated by electrical cables during operation dissipates through the duct and surrounding encasement concrete into the adjacent soil. Consequently, the thermal conductivity of the encasement concrete is critical for the overall heat dissipation efficiency and thermal stability of the cable system. To address this, a high-thermal-conductivity concrete was developed by incorporating steel slag powder, graphite powder, and graphite particles. This study systematically investigated the effects of mineral admixture content, graphite powder dosage, and the replacement ratio of conductive aggregates on the thermal conductivity, mechanical properties, and workability of the concrete. Additionally, the economic performance was evaluated against conventional C25 concrete using value engineering methodology. The results demonstrate that the proposed concrete exhibited significantly improved thermal performance compared to ordinary concrete. Notably, with a 10% replacement of aggregates by graphite particles, the 28-day thermal conductivity increased by 106.16% relative to the control mix. Simultaneously, the compressive strength reached 25.1 MPa, ensuring sufficient mechanical integrity. Furthermore, the value coefficient of the developed concrete was 18.31% higher than that of conventional C25 concrete. These findings highlight the material’s potential in power engineering and energy-efficient construction, providing a valuable reference for material design in these fields.

## 1. Introduction

Cable duct banks are widely employed as protective structures for underground cable installation in power transmission systems [1]. During operation, underground power cables generate substantial heat, typically on the order of 20–60 W/m per cable depending on current load and insulation conditions, and inadequate heat dissipation can lead to insulation degradation, thermal breakdown, and even fire hazards. To mitigate these thermal risks and ensure safe and stable power system operation, measures such as current derating are typically adopted [2].

**Figure 1:**
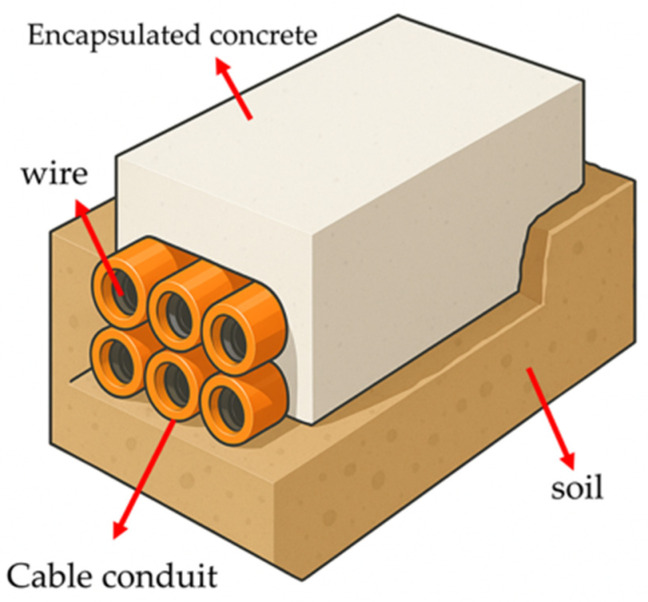
Schematic diagram of underground cable duct system.

A typical underground cable duct system comprises conductors, cable ducts, encasing concrete, and surrounding soil, as illustrated in Figure 1. From a heat transfer perspective, the heat generated during cable operation is first conducted through the ducts to the encasing concrete and subsequently dissipated into the surrounding soil. As the primary heat transfer medium, the thermal conductivity of concrete is a critical factor governing the efficiency and stability of heat dissipation. Therefore, developing high thermal conductivity concrete is of significant engineering importance for enhancing power transmission efficiency and ensuring system safety. Against this background, the present study aims to contribute to the existing literature by providing a comparative and engineering-oriented assessment of thermal conductivity and associated mechanical performance under controlled and consistent testing conditions.

The thermal conductivity of concrete has been extensively investigated due to its critical role in energy efficiency, underground infrastructure, and thermal management applications. Previous studies have shown that thermal conductivity is strongly influenced by multiple factors, including aggregate type and content [3], moisture condition [4], density and porosity [5], and testing methodology [6]. In particular, the presence of moisture within the pore structure can significantly increase heat transfer efficiency, often resulting in thermal conductivity values markedly higher than those measured under dry or air-dried conditions. Previous studies by Chung and co-workers at the University at Buffalo have extensively investigated the incorporation of graphite and other carbon-based fillers in cementitious materials, with particular emphasis on electrical, mechanical, and multifunctional properties. These studies have provided valuable insights into the role of graphite on transport behavior and microstructural connectivity. In contrast, the present study focuses specifically on the thermal conductivity enhancement under conditions relevant to underground power cable backfill materials, with an emphasis on the coupled effects of graphite and steel slag on thermal transport performance [7].

Several researchers have explored material modification strategies to enhance the thermal conductivity of concrete, such as incorporating high-conductivity aggregates, industrial by-products, or carbon-based additives [8]. While these approaches can improve heat transfer performance, they are frequently accompanied by changes in mechanical properties, workability, and durability, highlighting the need for balanced performance assessment rather than isolated optimization of thermal properties. Despite the growing body of literature on thermally enhanced concrete, many existing studies have focused on a limited number of material variables or specific additive systems, often under differing moisture conditions and testing protocols. As a result, direct comparison among different modification strategies remains challenging. There is a need for comparative and parametric investigations conducted under consistent experimental conditions to clarify relative performance trends and material trade-offs relevant to engineering applications.

From a materials composition perspective, both supplementary cementitious materials (SCMs) and aggregates significantly influence the thermal conductivity of concrete. Regarding SCMs, their type and proportion play a key role in tailoring thermal performance. Du et al. [9] reported a 170% increase in the 7-day thermal conductivity when 60% of cement was replaced with silicon carbide (SiC) powder. Yuan et al. [10] achieved a 196.5% improvement in the thermal conductivity of cement paste by incorporating graphite powder. Song et al. [11] demonstrated that cement mortars containing 20% iron powder and 20% copper powder exhibited thermal conductivities 19% and 29% higher than the control group, respectively. Graphite powder possesses an exceptionally high thermal conductivity (~200 W/(m·K)), far exceeding that of cement (~0.5–1 W/(m·K)), and forms thermally conductive pathways within the cement matrix, facilitating rapid heat transfer. Steel slag powder, a hard mineral admixture with excellent filler effect, enhances paste density and reduces air voids—given that air has a thermal conductivity of only 0.025 W/(m·K)—thereby effectively improving the overall thermal performance. Furthermore, as an industrial by-product, steel slag offers low cost and high reuse value, making it a preferred choice in sustainable construction materials.

Regarding aggregate selection, Woo et al. [12] replaced 50% and 100% of fine aggregates with silicon carbide and incorporated 1 vol.% steel fibers and 5 vol.% graphite particles, achieving a 1.78–2.47-fold increase in thermal conductivity. Yi et al. [13] introduced granite into backfill materials and observed a continuous increase in thermal conductivity with granite content; at 50% replacement, the thermal conductivity improved by approximately 26.7% compared to the reference group. Xing et al. [14] reported that substituting limestone aggregates with quartz aggregates increased the room-temperature thermal conductivity of concrete by approximately 1.7–2.0 times. Quartz sand exhibits a relatively high thermal conductivity (6–8 W/(m·K)) and favorable particle gradation, contributing to the continuity of the thermal network. Granite, a polycrystalline mineral composed of quartz, feldspar, and mica, has a higher thermal conductivity than typical natural aggregates; when used as coarse aggregate distributed throughout the concrete macrostructure, it helps form a meso-scale thermally conductive framework.

In concrete, heat is primarily conducted through the solid skeleton, while air voids act as the most significant thermal barriers. Replacing portions of the low-conductivity phases (paste or aggregates) with high-conductivity phases (e.g., quartz sand, granite, graphite, or steel slag) can substantially enhance the overall thermal conductivity. Consequently, the selection of high thermal conductivity raw materials is fundamental to developing high thermal conductivity concrete. This study systematically investigated the effects of common mineral admixtures (fly ash, steel slag, and ground granulated blast-furnace slag), graphite powder, and partial replacement of fine aggregates with graphite particles on thermal conductivity, workability, and mechanical properties. Additionally, a value engineering approach [15,16] was employed to evaluate the economic viability of the developed high thermal conductivity concrete compared to conventional C25 concrete. A high thermal conductivity concrete formulation was developed using steel slag and graphite powder as admixtures, graphite particles as partial replacement for quartz sand fine aggregates, and granite as coarse aggregate, providing a reference for the design of high thermal conductivity concrete materials. Accordingly, this work is positioned as a comparative and parametric experimental study aimed at revealing material-level performance trends, rather than a comprehensive optimization or finalized material design investigation.

Research objectives and scope: The objective of this study is to provide a systematic and engineering-oriented assessment of high thermal conductivity concrete designed for underground cable duct bank encasement. Rather than optimizing a single mix composition or conducting a comprehensive life-cycle comparative engineering cost assessment, this work focuses on clarifying the relative performance trends and trade-offs among thermal conductivity, mechanical properties, workability, and material-level cost.

Specifically, the study is structured around three progressive research questions: (i) how different mineral admixtures influence the thermal and mechanical behavior of cementitious matrices; (ii) how graphite powder content affects the balance between thermal enhancement and strength degradation; and (iii) how partial replacement of fine aggregates with graphite particles contributes to the formation of an effective thermal conduction network at the concrete scale. Based on these investigations, a representative high thermal conductivity concrete mixture was developed and evaluated using a comparative value engineering approach against conventional C25 concrete.

Within this defined scope, the comparative engineering cost assessment is intended to support material comparison rather than absolute cost prediction, and the experimental results are interpreted as laboratory-scale performance trends relevant to cable duct bank applications.

## 2. Materials and Methods

### 2.1. Materials

Portland cement (P.O 42.5) was supplied by Huaxin Cement Co., Ltd., Wuhan, Hubei, China, and its properties complied with the Chinese standard GB 175-2023 [17] Common Portland Cement. The physical properties of the cement are summarized in Table 1.

Steel slag was provided by Henan Rongsong Construction Engineering Co., Ltd., Zhengzhou, China. Ground granulated blast-furnace slag (GGBFS) and fly ash were obtained from the Qingshan District Steel Plant, Wuhan, China. Graphite powder and graphite particles were supplied by Zhengzhou Jingguo Graphite Co., Ltd., Zhengzhou, China. Quartz sand was produced by Zhengzhou Runjia Water Purification Materials Co., Ltd., Zhengzhou, China. Granite coarse aggregate was sourced from a quarry in the Yichang region, Hubei Province. A polycarboxylate-based superplasticizer, provided by a local cement plant in Yichang, was used to adjust workability. Tap water from the laboratory was used for mixing. The main chemical compositions of the raw materials are presented in Table 2.

### 2.2. Method

#### 2.2.1. Preparation Method

To address the research objectives outlined above, a three-stage progressive experimental program was designed, in which material composition was gradually refined from cement paste to concrete scale to clarify the individual and combined effects of mineral admixtures, graphite powder, and thermally conductive aggregates.

- Mineral admixtures

As shown in Table 3, the water-to-binder ratio (w/b) was maintained at 0.35. Cement was partially replaced by steel slag (S), ground granulated blast-furnace slag (G), and fly ash (F) to investigate the effects of different mineral admixtures on material properties. The replacement levels for each admixture were 10%, 20%, and 30% by mass of cement.

- Graphite powder

As shown in Table 4, the water-to-binder ratio was maintained at 0.35, with a steel slag content of 20% by mass of cement. Graphite powder (C) was used to partially replace cement to evaluate its effects on material properties. The replacement levels of graphite powder were 1%, 2%, 3%, 4%, 5%, 6%, and 7% by mass of cement.

- High thermal conductivity aggregates

As shown in Table 5, the water-to-cement ratio (w/c) was set at 0.45, with a sand ratio of 0.40. The steel slag content was 20% by mass of cement, and the graphite powder content was 5% by mass of cement. Granite was used as the coarse aggregate, while the fine aggregate consisted of quartz sand and graphite particles (GP). Graphite particles were used to partially replace quartz sand at replacement levels of 0%, 5%, 10%, and 15% by mass of quartz sand. A polycarboxylate-based superplasticizer was incorporated to maintain slump values above 160 mm for all mixtures.

#### 2.2.2. Test Methods

1. Chemical composition analysis

The chemical compositions of raw materials were determined using a Zetium X-ray fluorescence (XRF) spectrometer (Malvern Panalytical, Almelo, The Netherlands) to ensure compliance with experimental design requirements.

2. Thermal conductivity testing

Thermal conductivity measurements were performed using a Hot Disk thermal constants analyzer (Hot Disk AB, Gothenburg, Sweden) in accordance with ISO 22007-2 [18]. During testing, ambient temperature and humidity were maintained at stable levels to ensure the accuracy and comparability of results. Prior to thermal conductivity testing, all specimens were cured under standard conditions and subsequently stored in a controlled laboratory environment at (20 ± 2) °C and relative humidity of (60 ± 5)% until mass stabilization was achieved. Thermal conductivity measurements were conducted under this stabilized moisture condition. Although the exact gravimetric moisture content was not directly measured, the specimens can be considered to be in a near-dry and equilibrium moisture state at the time of testing.

3. Mechanical properties testing

Compressive strength tests were conducted in accordance with the Chinese standard GB/T 50081-2019 [19] Test Methods of Physical and Mechanical Properties of Concrete, using a WAY-300 fully automatic flexural and compressive testing machine (Wuxi Xiyi Building Materials Instrument Factory, Wuxi, China). Specimens were tested at curing ages of 3, 7, and 28 days under a loading rate of 0.4 MPa/s. Three parallel specimens were tested for each mix proportion, and the average value was reported as the compressive strength.

4. Workability testing

The fluidity of cement paste was measured according to the Chinese standard GB/T 2419-2023 [20] Test Method for Fluidity of Cement Mortar.

5. Value engineering method

In the value engineering analysis, the value coefficient V was employed to characterize the relationship between functionality and cost, defined as Equation (1) [16]:

(1) $$V=\frac{F}{C}$$

where F is the function coefficient representing the weighted comprehensive evaluation of material performance, and C is the cost coefficient representing the total cost per unit volume of concrete.

The function coefficient F is calculated using Equation (2):

(2) $$F=\sum_{i=1}^{n}(s_{i}\times \rho_{i})$$

where $s_{i}$ is the score of the concrete material for the i-th functional index, and $\rho_{i}$ is the weight of the i-th functional index.

Based on the requirements of cable duct bank encasing engineering, four functional indices were selected: thermal performance (*Q*_1_), mechanical performance (*Q*_2_), workability (*Q*_3_), and environmental sustainability (*Q*_4_). The analytic hierarchy process (AHP) [16] was employed to determine the weights of these indices. A judgment matrix A was established based on the relative importance of each index. Assuming that the relative importance of indices *Q*_1_, *Q*_2_, *Q*_3_, …, *Q_n_* with respect to the objective O is denoted as $P_{ij}$, where $P_{ij}=Q_{i}/Q_{j}$, the judgment matrix is expressed as $A=[P_{ij}{]}_{n\times n}$, satisfying $P_{ij}=1/P_{ji}$ and $P_{ji}>0$ for i,j=1,2,…,n.

To ensure the reliability of the judgment scale $P_{ij}$, domain experts were invited to conduct pairwise comparisons using a 1–9 scale [16], where intermediate values (2, 4, 6, 8) indicate intermediate importance levels, and reciprocal values (1/2, 1/3, …, 1/9) indicate inverse relationships. The judgment scale is presented in Table 6.

The judgment matrix was constructed as shown in Equation (3):

(3) $$A=\left[\begin{array}{cccc} 1 & 3 & 5 & 7\\ 1/3 & 1 & 3 & 5\\ 1/5 & 1/3 & 1 & 3\\ 1/7 & 1/5 & 1/3 & 1 \end{array}\right]$$

The judgment matrix A was normalized by columns to obtain matrix $B=[b_{ij}{]}_{n\times n}$, as shown in Equation (4):

(4) $$b_{ij}=\frac{P_{ij}}{\sum_{i=1}^{n}P_{ij}},i,j=1,2,\ldots ,n$$

The weight of each functional index $\rho_{i}$ was then calculated by averaging each row, as shown in Equation (5):

(5) $$\rho_{i}=\frac{1}{n}\sum_{j=1}^{n}b_{ij}$$

The consistency of the judgment matrix was verified by calculating the maximum eigenvalue ($\lambda_{max}\approx 4.118$), the consistency index (CI≈0.0395), and the consistency ratio (CR≈0.0439). Since CR<0.1, judgment matrix A demonstrated satisfactory consistency.

The resulting weights for the functional indices were determined as follows: thermal performance (*Q*_1_) = 0.558, mechanical performance (*Q*_2_) = 0.263, workability (*Q*_3_) = 0.122, and environmental sustainability (*Q*_4_) = 0.057.

The cost coefficient C represents the total cost per cubic meter of concrete, comprising material cost, labor cost, equipment cost, and management cost, as defined in Equation (6):

(6) $$C=C_{m}+C_{l}+C_{e}+C_{i}$$

where $C_{m}$, $C_{l}$, $C_{e}$, and $C_{i}$ represent the costs of materials, labor, equipment, and management, respectively.

6. Experimental repeatability and data analysis

For each concrete mixture, multiple specimens were prepared and tested to ensure measurement repeatability. Thermal conductivity measurements were conducted on three independently prepared specimens for each mix, and each specimen was measured three times under identical conditions. The reported thermal conductivity values represent the average of these measurements.

Compressive strength tests were performed on three cubic specimens for each mixture in accordance with the corresponding testing standards. The mean values are reported in this study. Measurement uncertainty associated with the thermal conductivity tester was within the manufacturer-specified accuracy range, and efforts were made to minimize systematic errors by maintaining consistent specimen dimensions, curing conditions, and testing procedures across all mixtures.

Basic statistical analysis was applied to the experimental results. Where applicable, standard deviations are provided to indicate data dispersion and measurement variability. Given the comparative and trend-focused objective of this study, no advanced statistical hypothesis testing was performed. This trend-focused approach is consistent with recent experimental studies on cement-based composites, where comparative analysis under standardized conditions is emphasized over formal hypothesis testing [9,21].

## 3. Results and Discussion

Unless otherwise stated, all experimental results presented in this section represent mean values obtained from repeated measurements, with observed variability remaining within an acceptable range for comparative analysis. While basic statistical descriptors were employed to assess data repeatability, the statistical analysis in this study was limited to descriptive statistics, consistent with the comparative and laboratory-scale nature of the investigation.

The moisture content of the specimens was not directly quantified at the time of thermal conductivity measurement. Future work should incorporate systematic control and measurement of moisture content to enable direct comparison with fully saturated or field-conditioned concrete and to support more comprehensive thermal performance modeling. It should be noted that moisture content is known to have a significant influence on the thermal conductivity of concrete [13,22]. In the present study, all mixtures were tested under comparable stabilized moisture conditions; therefore, the reported results are intended to reflect relative performance differences rather than absolute thermal conductivity values under varying moisture states.

### 3.1. Effects of Mineral Admixtures

**Figure 2:**
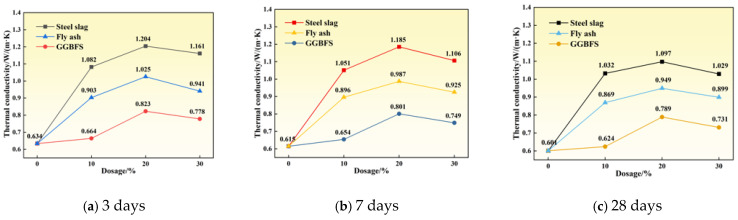
Variation of thermal conductivity of cement paste with age under different mineral admixture dosages at different curing ages.

Thermal conductivity is a critical parameter for evaluating the heat transfer performance of concrete, with higher values indicating superior thermal conduction and heat dissipation capabilities. Figure 2 presents the thermal conductivity of specimens incorporating different mineral admixtures at various curing ages. Overall, thermal conductivity exhibited a trend of initial increase followed by decrease with increasing admixture content, while decreasing gradually with extended curing age.

It is worth noting that the literature values correspond to different moisture contents, material compositions, and testing methods, and are only suitable for qualitative comparison. Specifically, at 3 days, the steel slag group with 20% replacement (S20) achieved the highest thermal conductivity of 1.204 W/(m·K), representing an 89.91% improvement over the reference group. The fly ash group (F20) and GGBFS group (G20) exhibited thermal conductivities of 1.025 W/(m·K) and 0.823 W/(m·K), respectively. At 7 and 28 days, the S20 group maintained thermal conductivities of 1.185 W/(m·K) and 1.097 W/(m·K), corresponding to improvements of 92.68% and 82.53%, respectively. These results demonstrate that an appropriate steel slag content can significantly enhance the thermal conductivity of concrete.

The observed trend of initial increase followed by subsequent decrease results from the combined effects of multiple factors. At replacement levels below approximately 20%, the proportion of unreacted particles remains limited, minimizing adverse effects on thermal performance. Under these conditions, the higher intrinsic thermal conductivity and appropriate fineness of steel slag and GGBFS optimize particle gradation, fill capillary and interconnected pores, enhance the density of the interfacial transition zone (ITZ), and establish a continuous solid-phase heat transfer network, thereby improving overall thermal conductivity [22]. When the replacement level exceeds 20%, the proportion of unreacted particles increases, the degree of hydration decreases, and solid-phase continuity is compromised. Simultaneously, the increased proportion of inert phases and pores interrupts thermal pathways and raises total porosity, resulting in reduced thermal conductivity.

The general decrease in thermal conductivity with extended curing age is primarily attributed to changes in moisture content and microstructural evolution. Continued hydration refines the pore structure and increases the proportion of closed pores, while free water is consumed and replaced by air, which has low thermal conductivity, thereby reducing the overall heat transfer capacity [23]. Furthermore, although late-stage hydration products enhance density, they also introduce fine pores and interfacial thermal resistance. Combined with drying shrinkage and microcracking effects, these factors further impede heat flow [24].

**Figure 3:**
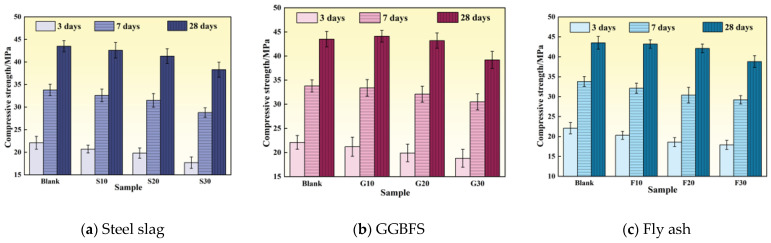
Variation of compressive strength of cement paste with age under different mineral admixture dosages.

Figure 3 illustrates the effects of different mineral admixtures on the compressive strength of concrete at various curing ages. Overall, compressive strength decreased with increasing replacement level. However, for GGBFS replacement, later-age strength exhibited an initial increase followed by a decrease. At 10% GGBFS content, the 28-day compressive strength reached 44.1 MPa, representing a 1.4% improvement over the reference group. At 30% steel slag content (S30), the compressive strengths at 3, 7, and 28 days were 17.7 MPa, 28.8 MPa, and 38.3 MPa, representing decreases of 24.86%, 17.36%, and 13.58% compared to the reference group, respectively. At 30% fly ash content (F30), the compressive strengths at 3, 7, and 28 days were 17.9 MPa, 29.2 MPa, and 38.8 MPa, corresponding to reductions of 4.2 MPa, 4.6 MPa, and 4.7 MPa, respectively.

The reduction in compressive strength following steel slag and fly ash replacement is primarily attributed to their chemical composition and low early-age reactivity. Steel slag contains limited alite content, abundant RO phases, and free CaO and MgO, which are unfavorable for hydration reactions [25]. Fly ash exhibits weak early-age reactivity, making it difficult to form a dense C–S–H gel framework at 3 and 7 days. Additionally, replacing cement clinker with these admixtures produces a dilution effect, reducing the proportion of reactive components and decreasing early-age hydration product formation, thereby resulting in significant strength reduction.

In contrast, low GGBFS content promotes later-age strength development [26]. GGBFS possesses latent pozzolanic activity and can react with Ca(OH)_2_ to generate additional C–S–H gel. Its higher fineness also provides nucleation sites that promote hydration. At appropriate replacement levels, the remaining clinker content maintains system alkalinity and Ca(OH)_2_ supply, thereby mitigating the dilution effect and promoting later-age strength gain. However, at excessive replacement levels, reduced clinker content limits hydration, resulting in strength reduction.

**Figure 4:**
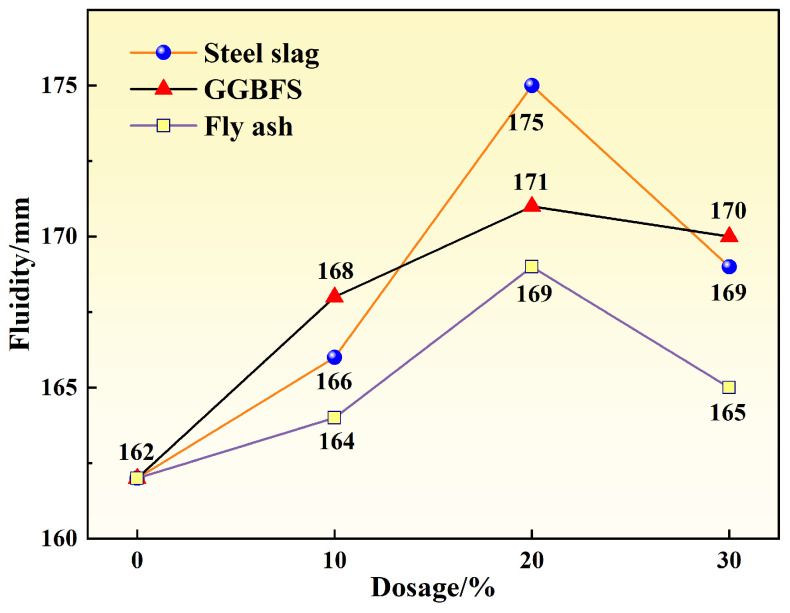
Fluidity of cement paste under different mineral admixture dosages.

As shown in Figure 4, the fluidity of specimens exhibited an initial increase, followed by a decrease with increasing mineral admixture content. At 20% replacement, the steel slag group (S20) demonstrated optimal fluidity, reaching 175 mm—an 8.02% improvement over the reference group. The GGBFS group (G20) followed with a fluidity of 171 mm. When the replacement level increased further to 30%, the fluidity of the steel slag group (S30) decreased to 169 mm, indicating reduced workability.

At low replacement levels, mineral admixtures with high particle roundness and moderate fineness produce a “ball-bearing effect” and micro-filling effect, which reduce interparticle sliding resistance and improve particle gradation, thereby enhancing paste fluidity [21]. When the replacement level exceeds approximately 20%, the total specific surface area of the system increases, and numerous fine particles adsorb free water and admixtures, reducing lubricating water and increasing water demand, which results in decreased fluidity. Simultaneously, at high replacement levels, the increased proportion of unreacted particles leads to a loose paste structure and increased internal friction. Consequently, concrete fluidity follows a pattern of enhancement at low replacement levels and deterioration at high replacement levels.

Within the investigated replacement range, specimens with 20% steel slag content exhibited the most balanced thermal, mechanical, and workability performance, making this level suitable for subsequent comparative investigations rather than representing a globally optimal composition.

### 3.2. Effects of Graphite Powder

**Figure 5:**
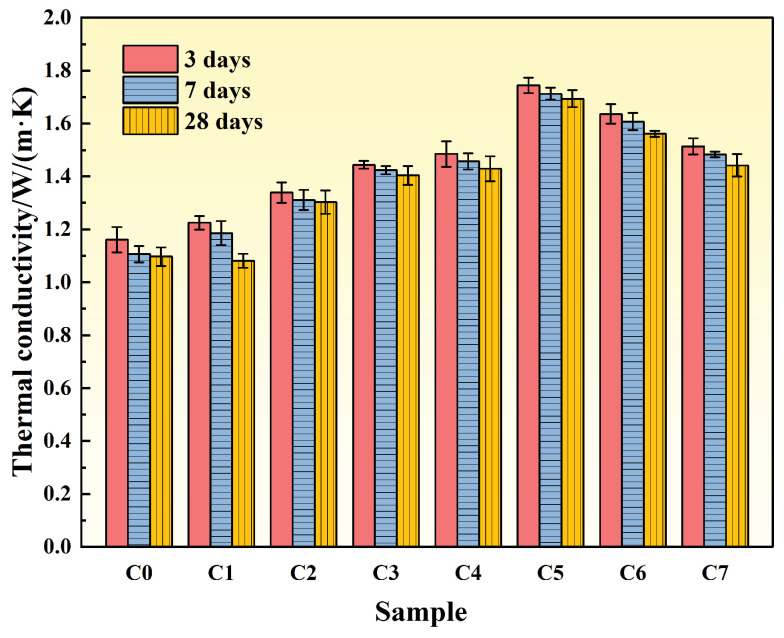
Variation of thermal conductivity of cement paste with age under different graphite powder contents (blank denotes the reference mixture without graphite powder).

Figure 5 illustrates the effects of graphite powder content on the thermal conductivity of specimens at various curing ages. Overall, the thermal conductivity exhibited a trend of initial increase followed by a decrease as graphite content increased, demonstrating a clear thermal enhancement effect. Concurrently, thermal conductivity gradually decreased with extended curing age. At 3 days, the thermal conductivity peaked at 1.744 W/(m·K) for the specimen with 5% graphite powder (C5), representing a 50.22% improvement over the reference group. When the content was increased to 7% (C7), the thermal conductivity decreased to 1.514 W/(m·K), a 15.19% reduction compared to the C5 specimen. At 7 and 28 days, the C5 specimen maintained the highest thermal conductivity at 1.712 W/(m·K) and 1.694 W/(m·K), corresponding to improvements of 54.79% and 54.42% over the reference group, respectively.

The observed trend of initial increase followed by subsequent decrease is attributed to the interplay of graphite’s high intrinsic thermal conductivity, its dispersion state, and interfacial heat transfer characteristics. Graphite possesses a layered crystal structure and an exceptionally high thermal conductivity (150–500 W/(m·K)). At an optimal content (e.g., 5%), graphite particles can be uniformly dispersed within the matrix, forming a continuous or semi-continuous thermal network. This network facilitates efficient heat transfer through point and surface contacts between particles, significantly reducing the system’s thermal resistance and enhancing overall thermal performance [27]. Conversely, when the graphite content exceeds the optimal range, particle agglomeration and poor interfacial bonding can occur, leading to increased porosity and interfacial thermal resistance, which has also been reported in recent studies on graphite-modified cement-based and composite materials [27,28]. Simultaneously, excessive graphite replaces a portion of the cement clinker, inhibiting hydration and reducing C–S–H gel formation, which weakens the matrix density and further compromises thermal conductivity [29].

The thermal conductivity values obtained in this study were generally lower than those reported in some previous studies for conventional concrete. This discrepancy should be interpreted in light of differences in moisture condition, material composition, and testing methodology. As widely reported in the literature, moisture content has a dominant influence on the thermal conductivity of concrete, and values measured under saturated or partially saturated conditions are often significantly higher than those obtained under near-dry states.

In the present study, all specimens were tested under stabilized, near-dry equilibrium moisture conditions. Under such conditions, the pore structure is predominantly air-filled, leading to reduced heat transfer efficiency compared to moisture-filled pores. Consequently, lower thermal conductivity values are expected and have been reported by other researchers investigating oven-dried or air-dried concrete specimens.

In addition, the incorporation of graphite powder and conductive aggregates in this study was limited to partial replacement levels designed to enhance thermal pathways without fundamentally altering the bulk density or pore structure of the concrete. The resulting thermal conductivity therefore reflects a balance between localized conductive enhancement and the overall lightweight and porous nature of the cementitious matrix. Differences in aggregate type, density, and testing techniques (e.g., transient versus steady-state methods) further contribute to variability among the reported literature values.

Accordingly, the thermal conductivity results presented in this study are not intended to provide absolute benchmark values for all service conditions, but rather to support a comparative evaluation of material modification strategies under consistent testing conditions.

**Figure 6:**
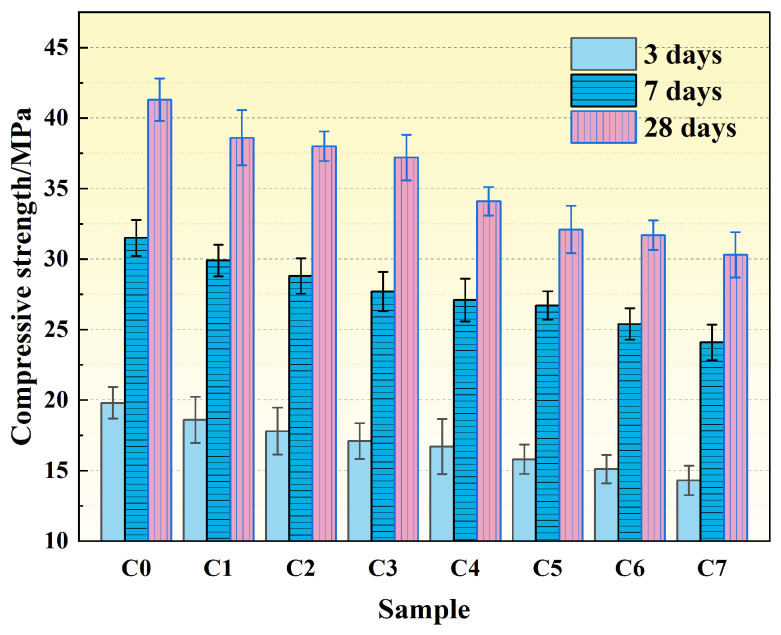
Variation of compressive strength of cement paste with age under different graphite powder contents.

Figure 6 shows the compressive strength results at different curing ages. The compressive strength of the cement paste specimens consistently decreased with increasing graphite powder content, with the reduction being particularly pronounced at early ages (3 days). Specifically, at a 5% graphite content, the 3-, 7-, and 28-day compressive strengths decreased by 25.31%, 17.98%, and 28.66%, respectively, compared to the reference group. When the content was increased to 7%, the 3-day strength reduction reached 38.46%. These findings indicate that the incorporation of graphite powder has a significant detrimental effect on the strength development of the cementitious system.

This strength reduction can be attributed to several mechanisms. First, as an inert filler, graphite powder does not participate in cement hydration, leading to a typical “dilution effect” by reducing the effective cement content. Second, the smooth and hydrophobic surface of graphite hinders the formation of a strong bond with the cement paste, resulting in a weak interfacial transition zone (ITZ) with localized pores and microcracks. This compromises the overall density and stress transfer capability of the matrix [30]. Third, due to its high specific surface area and lamellar structure, graphite is prone to agglomeration within the cement paste, creating weak zones that disrupt structural continuity [31]. Furthermore, the hydrophobicity of graphite inhibits the cement hydration process, thereby reducing the formation of early-age hydration products and causing the observed significant decrease in early strength.

**Figure 7:**
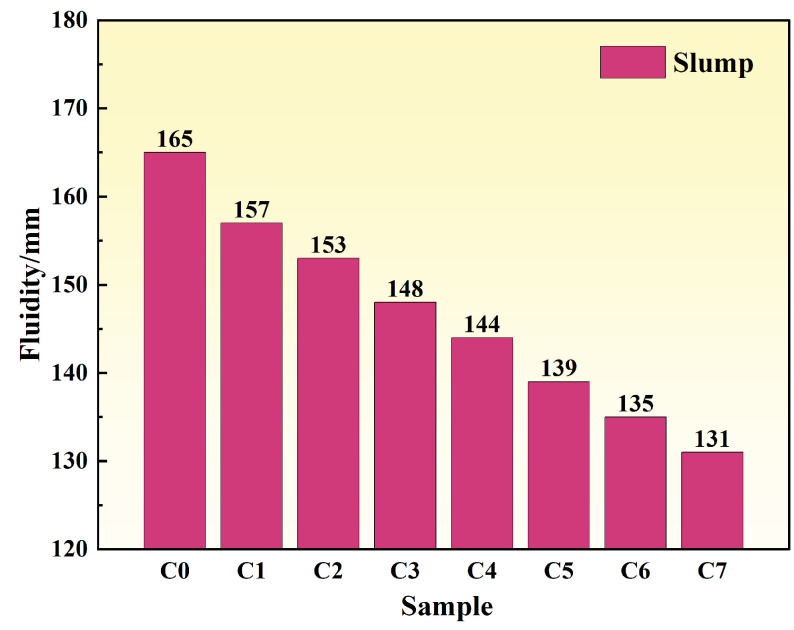
Fluidity of cement paste with different graphite powder dosages.

As shown in Figure 7, the fluidity of the cement paste decreased significantly with increasing graphite powder content, indicating a progressive reduction in workability. The reference group (C0) had a fluidity of 165 mm. When the graphite content was increased to 5% (C5), the fluidity dropped to 139 mm, an 18.71% decrease from the reference. At 7% content (C7), the fluidity further decreased to 131 mm, representing a 25.95% reduction. The decline in fluidity is primarily due to a combination of factors: the high specific surface area of graphite, which adsorbs free water; the interlocking effect of its platelet-like structure, which increases internal friction; and adverse interfacial effects [31].

As the graphite powder content increases, thermal conductivity shows a consistent upward trend, accompanied by a gradual reduction in compressive strength and workability. In the investigated dosage range, the results clarify the directional influence of graphite addition and serve to delineate performance boundaries for comparative analysis, rather than to identify a single optimal content.

### 3.3. Effects of High Thermal Conductivity Aggregates

**Figure 8:**
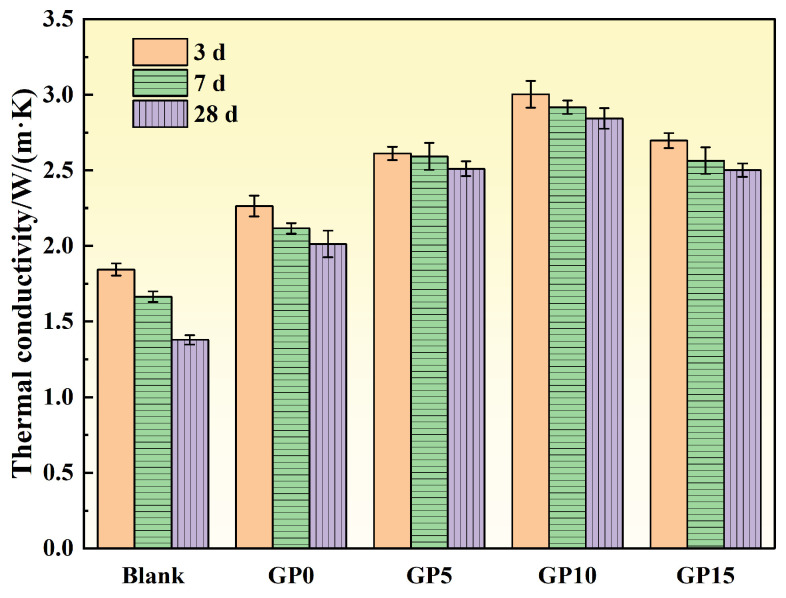
Variation of thermal conductivity of concrete with age under different graphite particle replacement rates.

Figure 8 illustrates that the thermal conductivity of the concrete exhibited a trend of initial increase followed by a decrease with increasing graphite particle replacement level. At low to moderate replacement levels (≤10%), thermal conductivity increased significantly. However, a further increase resulted in a decrease, suggesting a competition between the thermal enhancement effect of graphite and the structural degradation it induces. Concurrently, the thermal conductivity of all groups decreased with extended curing age, reflecting the time-dependent effects of pore structure evolution and hydration product distribution.

The 3-, 7-, and 28-day thermal conductivities of the control group (Blank) were 1.844, 1.665, and 1.379 W/(m·K), respectively. For the reference concrete containing high-performance aggregates but no graphite particles (GP0), these values were 2.264, 2.117, and 2.013 W/(m·K). At a 10% replacement level (GP10), the thermal conductivity peaked, reaching 3.003, 2.917, and 2.843 W/(m·K) at 3, 7, and 28 days, respectively. These values represent significant improvements of 62.85%, 75.19%, and 106.16% compared to the Blank group.

The enhancement in thermal conductivity is attributed to the formation of an efficient heat transfer network. The term ‘thermal network’ is used here in a descriptive sense to denote enhanced particle connectivity rather than a strict percolation transition. While a critical threshold is not explicitly identified, the observed optimum at approximately 10% graphite particle replacement suggests a connectivity-dominated enhancement regime. When graphite particles are uniformly dispersed, their highly crystalline, layered structure facilitates the formation of a continuous, high-conductivity phase network within the matrix. Heat transfer in the composite occurs primarily through phonon conduction within the graphite layers, complemented by solid–solid conduction through aggregates, interfacial heat transfer across the ITZ, and limited conduction through pore fluids [32]. At low to moderate replacement levels, the proximity of graphite particles allows for the interconnection of thermal bridges, improving the integrity of the thermal pathways and leading to a marked increase in thermal conductivity. Conversely, when the replacement level was increased to 15% (GP15), a decrease in thermal conductivity was observed. The hydrophobic surface of graphite leads to poor adhesion with the cement paste, creating distinct interfacial separation layers. These layers introduce high interfacial thermal resistance, which acts as a bottleneck for heat flow, disrupting the continuity of the thermal network and thereby reducing overall thermal efficiency [28].

In the early stages, the system contains a significant amount of free water, which has a relatively high thermal conductivity. As hydration progresses, this water is consumed and replaced by C–S–H gel and air-filled pores. Since air is a thermal insulator, the increasing complexity of the heat transfer path and the higher proportion of low-conductivity phases lead to a reduction in the overall thermal conductivity.

**Figure 9:**
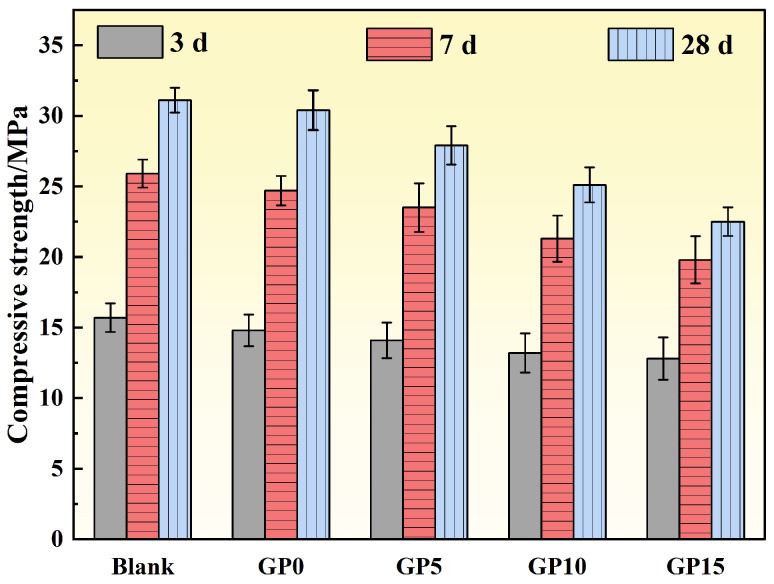
Variation of concrete compressive strength with age under different graphite particle replacement rates.

As shown in Figure 9, the compressive strength of the concrete at 3, 7, and 28 days consistently decreased with increasing graphite particle replacement level. The 3-, 7-, and 28-day compressive strengths of the Blank group were 15.7, 25.9, and 31.1 MPa, respectively. For the GP0 group, the corresponding strengths were 14.8, 24.7, and 30.4 MPa. At a 10% replacement level (GP10), the strengths decreased to 13.2, 21.3, and 25.1 MPa, which were 18.94%, 21.59%, and 23.91% lower than the Blank group. At a 15% replacement level (GP15), the strength reduction was even more pronounced, with the 28-day strength dropping to just 22.5 MPa. This indicates that excessive graphite content significantly impairs the load-bearing capacity of the concrete. Similar strength levels (20–30 MPa) have been reported to be acceptable for non-load-bearing underground infrastructure materials, where thermal and durability performance are prioritized over high structural strength [26].

The incorporation of graphite particles modifies heat transfer behavior at the concrete scale by promoting a more continuous conduction pathway, which explains the observed enhancement in thermal conductivity. Based on these trends, a representative mixture was selected for subsequent value engineering evaluation. When the replacement level becomes excessive, this benefit is offset by strength and workability losses, indicating that the results should be interpreted as comparative performance trends instead of a determination of an optimal aggregate replacement ratio.

### 3.4. Comparative Engineering Cost Assessment Using Value Engineering

The value engineering approach adopted in this study aims to support comparative material selection at the conceptual design stage. The criterion weights were determined based on expert judgment to reflect typical engineering priorities for underground cable duct applications, rather than to provide a universally applicable or market-optimized economic evaluation.

In cable duct bank encasement projects, selecting a concrete that effectively balances performance with economic viability is crucial for ensuring both project profitability and long-term operational safety. To facilitate a systematic material selection, a comparative engineering cost assessment was conducted using the value engineering method. This analysis compares the developed high-thermal-conductivity concrete (HTCC) with traditional C25 concrete to determine the optimal solution based on engineering costs.

As shown in Table 7, domain experts scored both the HTCC and conventional C25 concrete on a 10-point scale for the four indices: thermal performance, mechanical performance, workability, and environmental sustainability. The average scores were then used to calculate the function coefficient (F) using Equation (2). The resulting function coefficient was 7.661 for the HTCC, significantly higher than the 5.079 calculated for the C25 concrete. This study does not include durability-related tests such as freeze–thaw resistance, sulfate attack, carbonation, or chloride penetration. Previous studies have shown that the long-term performance of steel slag and GGBFS-containing cementitious materials is strongly dependent on mix design, curing regime, and exposure conditions [25,26]. Therefore, durability assessment is identified as an important direction for future work.

The unit costs for both concrete types were determined based on national construction cost standards and current market prices from material suppliers. The detailed material costs for the HTCC are listed in Table 8. As summarized in Table 9**,** the total cost coefficient (C), calculated using Equation (6), was determined to be 911 for the HTCC and 715 for the conventional C25 concrete. The optimal solution is shown in Table 10.

## 4. Conclusions

Within the defined comparative and laboratory-scale scope of this study, the experimental results and value engineering analysis led to the following conclusions:

(1) The incorporation of 20% steel slag as a partial cement replacement effectively enhanced the thermal conductivity of the specimens while maintaining satisfactory mechanical performance. Specifically, it increased the 28-day thermal conductivity to 1.097 W/(m·K), an 82.53% improvement over the reference group, and achieved a 28-day compressive strength of 38.3 MPa.
(2) The addition of graphite powder proved to be an effective strategy for enhancing thermal conductivity. An optimal content of 5% resulted in a 28-day thermal conductivity of 1.694 W/(m·K), marking a 54.42% improvement compared to the reference group, though it adversely affected the mechanical strength and workability.
(3) In the development of the high thermal conductivity concrete, partially replacing quartz sand with 10% graphite particles (by volume of fine aggregate) resulted in a remarkable enhancement of the concrete’s thermal performance. This modification increased the 28-day thermal conductivity by 106.16% compared to the control group.
(4) The value engineering analysis demonstrated that the developed high thermal conductivity concrete (HTCC) possesses superior economic viability. With a value coefficient 18.31% higher than that of conventional C25 concrete, the HTCC presents a more cost-effective solution with significant potential for practical engineering applications.

## Figures and Tables

**Table 1:** Physical properties of cement.

Specific Surface Area/(m^2^/kg)	Apparent Density/(kg/m^3^)	Loss on Ignition/%	Initial Setting Time/min	Final Setting Time/min
352	3110	3.02	93	261

**Table 2:** Main chemical components of raw materials (wt.%).

Material	CaO	Al_2_O_3_	SiO_2_	Fe_2_O_3_	SO_3_	MgO	C	Loss
Cement	62.58	4.29	20.75	3.57	2.17	1.46	—	1.21
Steel slag	32.04	5.88	19.57	26.19	0.77	4.81	—	6.24
GGBFS	33.71	11.42	33.5	1.09	1.13	4.05	—	5.71
Fly ash	3.11	25.61	50.82	6.17	1.01	0.51	—	3.87
Graphite powder	—	—	—	—	—	—	99.25	0.05

**Table 3:** Mix design of cement paste with different mineral admixture dosages.

Sample	Cement/g	Steel Slag/g	GGBFS/g	Fly Ash/g	Water/g
Blank	300	0	0	0	105
S10	270	30	0	0	105
S20	240	60	0	0	105
S30	210	90	0	0	105
G10	270	0	30	0	105
G20	240	0	60	0	105
G30	210	0	90	0	105
F10	270	0	0	30	105
F20	240	0	0	60	105
F30	210	0	0	90	105

**Table 4:** Mix design of cement paste with different graphite powder dosages.

Sample	Cement/g	Steel Slag/g	Graphite Powder/g	Water/g
C0	240	60	0	105
C1	237	60	3	105
C2	234	60	6	105
C3	231	60	9	105
C4	228	60	12	105
C5	225	60	15	105
C6	222	60	18	105
C7	219	60	21	105

**Table 5:** Mix design of HTCC (kg/m^3^).

Sample	Cement	Steel Slag	Graphite Powder	River Sand	Quartz Sand	Graphite Particles	Granite	Water
Blank	460	-	-	720	-	-	1080	207
GP0	345	92	23	-	720	0	1080	207
GP5	345	92	23	-	684	36	1080	207
GP10	345	92	23	-	648	72	1080	207
GP15	345	92	23	-	612	108	1080	207

**Table 6:** Judgment criteria.

*P_ij_*	1	2	3	4	5	6	7	8	9
The importance of $\frac{Q_{i}}{Q_{j}}$	Equally important	Between 1 and 3	Slightly important	Between 3 and 5	Significantly important	Between 5 and 7	Strongly important	Between 7 and 9	Extremely important

**Table 7:** Performance rating of concrete materials under different indicators (out of 10 points).

Research Subjects	Thermal Conductivity	Mechanical Properties	Performance	Environmental Protection
C25 ordinary concrete	3.33	7.67	8	4
HTCC	8.67	7.33	5	5

**Table 8:** Cost of HTCC materials.

Material	Dosage (kg/m^3^)	Unit Cost (Yuan/kg)	Material Cost (Yuan)
Cement	345	0.4	138
Steel Slag	92	0.12	11.04
Graphite Powder	23	4	92
Water	207	0.0095	1.96
Quartz Sand	648	0.1	64.8
Graphite Particles	72	3	216
Granite	1080	0.09	97.2
PCE	1.5	10	15
Total	-	-	636

**Table 9:** Cost coefficient.

Cost	C25 Ordinary Concrete	HTCC
Material Cost	440	636
Labor Cost	90	90
Equipment Cost	65	65
Overhead Cost	120	120
Cost Coefficient C	715	911

**Table 10:** Value coefficient.

Alternative	Function Coefficient (F)	Cost Coefficient (C)	Value Index (V)	Optimal Alternative
C25 ordinary concrete	5.079	715	0.0071	
HTCC	7.661	911	0.0084	▲

“▲” indicates the optimal solution (HTCC) compared to the C25 reference concrete.

## Data Availability

The original contributions presented in this study are included in the article. Further inquiries can be directed to the corresponding author.

## Abbreviations

The following abbreviations are used in this manuscript:

HTCC	High thermal conductivity concrete
ITZ	Interfacial Transition Zone
GGBFS	Ground Granulated Blast-Furnace Slag
w/b	Water-to-binder ratio
w/c	Water-to-cement ratio
AHP	Analytic Hierarchy Process
XRF	X-ray Fluorescence
PCE	Polycarboxylate Superplasticizer
